# Mangiferin ameliorates polycystic ovary syndrome in rats by modulating insulin resistance, gut microbiota, and ovarian cell apoptosis

**DOI:** 10.3389/fphar.2024.1457467

**Published:** 2024-09-23

**Authors:** Zhang Yong, Chen Mimi, Li Yingjie, Guo Yichen, Yu Yansu, Zhou Zhi, Lu Hui, Yao Si, Wu Chongming, Zhang Xiaopo, Ma Ning, Lu Weiying

**Affiliations:** ^1^ Key Laboratory of Tropical Translational Medicine of Ministry of Education and Department of Pharmacology, School of Basic Medicine and Life Sciences, Hainan Medical University, Haikou, China; ^2^ Hainan Academy of Medical Sciences, Hainan Medical University, Haikou, China; ^3^ School of Pharmacy, Harbin University of Commerce, Harbin, China; ^4^ Reproductive Medical Center, Hainan Women and Children’s Medical Center, Haikou, China; ^5^ School of Chinese Materia Medica, Tianjin University of Traditional Chinese Medicine, Tianjin, China; ^6^ Haikou Key Laboratory of Li Nationality Medicine, Hainan Key Laboratory for Research and Development of Tropical Herbs, Engineering Research Center of Tropical Medicine lnnovation and Transformation of Ministry of Education and International Joint Research Center of Human-Machine Intelligent Collaborative for Tumor Precision Diagnosis and Treatment of Hainan Province, School of Pharmacy, Hainan Academy of Medical Sciences, Hainan Medical University, Haikou, China

**Keywords:** mangiferin, PCOS, hormonal imbalance, insulin resistance, gut microbiota, ovarian cell apoptosis, female rats

## Abstract

Polycystic ovary syndrome (PCOS) is a complex endocrine and metabolic disorder characterized by hyperandrogenism, prolonged anovulation and polycystic ovaries. However, there are no effective interventions to treat this disorder. As previously shown, mangiferin modulated the AMPK and NLRP3 signal pathways to alleviate nonalcoholic fatty liver disease (NAFLD). In recent years, mangiferin has emerged as a promising drug candidate for treating metabolic diseases. In this study, we evaluated the effects of mangiferin on a letrozole (LET) combined with high-fat diet (HFD)-induced PCOS rat model through estrous cycle detection, serum/tissue biochemical analysis, and hematoxylin and eosin (HE) staining of ovarian tissue. The mechanisms of mangiferin’s effects on PCOS rats were analyzed using 16S rRNA sequencing, RNA-seq, western blotting (WB), and immunohistochemical (IHC) staining. Our results displayed that mangiferin showed a promising effect in PCOS rats. It improved lipid metabolism, glucose tolerance, insulin resistance, hormonal imbalance, ovarian dysfunction, and adipocyte abnormalities. RNA-seq analysis indicated that mangiferin may be involved in several signal pathways, including apoptosis, necrosis, and inflammation. Furthermore, western blot and immunohistochemical staining demonstrated that mangiferin regulates Caspase-3 and Cytc, exhibiting anti-apoptotic activity in the ovaries. Additionally, mangiferin significantly altered the gut microbiota community of PCOS rats, changing the abundance of firmicutes, bacteroidota, proteobacteria, and actinobacteria at the phylum level and the abundance of *Blautia*, *Coprococcus*, *Roseburia,* and *Pseudomonas* at the genus level. In conclusion, mangiferin is a promising and novel therapeutic agent for PCOS as it ameliorates insulin resistance, gut microbiota and ovarian cell apoptosis.

## 1 Introduction

Polycystic ovary syndrome (PCOS) is one of the most common endocrine and metabolic disorders affecting 5%–20% of women of reproductive age worldwide (Heinonen et al., 2020; Stener-Victorin et al., 2020). Historically, PCOS was considered a disorder in adult women. However, recent researches have revealed that PCOS is a lifelong condition (Di Lorenzo et al., 2023). The main clinical manifestations of PCOS include hyperandrogenism, chronic anovulation and polycystic ovaries (Armanini et al., 2022; Zong et al., 2019; Xu et al., 2023). The high prevalence of PCOS and its association with impaired ovulation and menstruation, infertility, and metabolic problems imposes an enormous burden on patients and society. Nonetheless, current treatments mainly focus on symptom relief, lacking effective etiological therapies (Xie et al., 2021; Motlagh Asghari et al., 2022; Gurule et al., 2023).

In recent years, increasing evidences have indicated that insulin resistance (IR) plays a crucial role in the pathogenesis of PCOS. Many patients with PCOS exhibit decreased insulin sensitivity, which exacerbates metabolic abnormalities and may further enhance hyperandrogenemia and ovarian dysfunction (Dunaif, 2020). Therefore, improving IR is a critical strategy for treating PCOS. The gut microbiota is instrumental in host metabolism and immune regulation. Studies have found significant differences in the gut microbiota structure between PCOS patients and healthy individuals, and these differences are closely related to IR and inflammatory responses (Torres et al., 2018). Modulating the gut microbiota may thus offer novel avenues for PCOS treatment. Ovarian cell apoptosis is also significant in the development of PCOS. Increased apoptosis rates in ovarian granulosa cells have been observed in PCOS patients, potentially leading to diminished ovarian function and impaired follicular development (Sen et al., 2014). Hence, inhibiting ovarian cell apoptosis may help improve reproductive function in PCOS patients.

Combined oral contraceptives, metformin, and other drugs have been recommended for the treatment of PCOS (Jelodar et al., 2018; Sathyapalan et al., 2019; Hoeger and Dokras, 2021). However, due to their side effects, their long-term clinical application is limited. Therefore, finding new safe and effective treatments for PCOS is crucial. An increasing number of studies have shown that natural products such as *Moringa oleifera* leaves, *L-carnitine*, and *Ginkgo biloba* can significantly alleviate PCOS-related symptoms by regulating IR, exerting antioxidant effects, and modulating gut microbiota (Iervolino et al., 2021; Nofal et al., 2024; Wu et al., 2023). Thus, natural products hold promise as potential therapeutic options for the treatment of polycystic ovary syndrome (PCOS).

Mangiferin, a compound extracted from *Mangifera indica* L., has also been found in plants belonging to the Liliaceae, Fabaceae, and Hypericaceae families (Hering et al., 2023). As a bioactive compound, studies have shown that mangiferin can regulate glucose and lipid metabolism, improve IR, exert anti-inflammatory effects, and modulate gut microbiota, providing a theoretical basis for its application in the treatment of polycystic ovary syndrome (PCOS) (Qian et al., 2020; Al-Saeedi, 2021). In a PCOS rat model induced by letrozole, mangiferin demonstrated significant protective effects. The research found that mangiferin improved ovarian structural damage and follicular dysplasia by reducing oxidative stress markers and pro-inflammatory factor expression. Its antioxidant and anti-inflammatory mechanisms play a key role in alleviating ovarian dysfunction caused by PCOS (Al-Saeedi, 2021). Given the reported biological activities of mangiferin, the present study aimed to further explore the effects and potential mechanisms of mangiferin in letrozole and high-fat diet (HFD)-induced PCOS models, providing new insights and evidence for PCOS treatment.

## 2 Materials and methods

### 2.1 Reagents

Mangiferin (PubChem CID: 5281647, Catalog Number: BP0922, Purity: ≥ 98%) was supplied by cdpurify (Chengdu, China). Metformin (PubChem CID: 4091, Catalog Number: S30880, Purity: ≥ 98) was supplied by shyuanye (Shanghai, China). A high-fat diet (HFD, Catalog Number: D12451 45 Kcal% Fat, future biotech). The triglyceride (TG, Cat Number: A110-1-1), total cholesterol (CHO, Cat Number: A111-1-1), and a low density lipoprotein-cholesterol (LDL, Cat Number: A113-1-1) assay kits were purchased from Nanjing Jian cheng Bioengineering Institute. The serum Testosterone (T, Cat Number: E-EL-0155), Luteinizing Hormone (LH, Cat Number: E-EL-R0026), Follicle Stimulating Hormone (FSH, Cat Number: E-EL-R0391), Insulin(Cat Number:E-EL-R3034), and Estradiol (E2, Cat Number: E-EL-0152) ELISA kits were purchased from Elabscience Biotechnology Co., Ltd.Anti-Cytc (Catalog Number: 10993-1-AP) was purchased from Proteintech Group, Inc. Anti-Caspase3 (Catalog Number: 9662S), anti-rabbit IgG (Catalog Number: 7074S) and anti-mouse IgG (Catalog Number: 7076S) were purchased from Cell Signalling Technology, Inc.

### 2.2 Animals and experimental design

A total of 30 three-week-old, pathogen-free SD female rats were purchased from Gempharmatech Co., Ltd. (Jiang Su, China). The rats were housed individually at a room temperature of 23°C ± 2°C, humidity of 55% ± 5%, and a 12-h light/dark cycle. After 1 week of acclimation, the rats were randomly divided into 2 groups: normal group (NC group, n = 6) and PCOS model group (n = 24). The NC group was fed a basic diet, while the PCOS model group was fed a high-fat diet (HFD). After 4 weeks, fasting blood glucose (FBG) and serum insulin levels were measured in all rats.

From week 5 to week 8, the PCOS model group continued to receive HFD and letrozole (1 mg/kg/d) injections, while the NC group received a basic diet and sterile saline injections as a control. After successful modeling, the PCOS model group was randomly divided into 4 groups (n = 6): PCOS model group, a low-dose mangiferin group (ML group) (50 mg/kg/d), a high-dose mangiferin group (MH group) (200 mg/kg/d), and metformin group (Met group) (200 mg/kg/d) (Zhang et al., 2021). The NC group received a normal diet, while the PCOS model group, ML group, MH group and Met group continued on HFD. Mangiferin was dissolved in vegetable oil, and metformin was dissolved in saline. Vaginal smears were collected twice in weeks 7 and 11, respectively. On the week 9 and week 13, FBG and serum insulin level were measured, respectively. All animals were anesthetized and euthanized on the first day of week 13. Samples from blood, liver, subcutaneous fat, ovarian fat, ovaries, wombs, and intestines were collected. Throughout the experiment, food intake and body weight changes were recorded. All animal facilities and protocols complied with the National Institutes of Health guidelines for the care and use of laboratory animals and were approved by the Hainan Medical University (No.: syxk-2017-0013).

### 2.3 Estrous cycle determination

The rats’ estrous cycle was assessed using a vaginal smear (Yang et al., 2019). A sterile cotton swab dipped in sterile saline was inserted into the rat’s vagina, gently rotated, and applied to the slide, which was then air-dried and stained methylene blue (MB). The estrous cycle stage was determined based on vaginal cytology.

### 2.4 Oral glucose tolerance test (OGTT)

After 12 h of fasting, rats were weighed, and blood glucose levels were measured before the injection. Glucose (0.2 g/mL) was injected intraperitoneally according to body weight (100 μL/10 g, injection volume to body weight ratio). Tail blood glucose levels were measured at 15, 30, 60, and 120 min after injection (Cao et al., 2022).

### 2.5 Insulin tolerance test (ITT)

After 6 h of fasting, rats were weighed, and blood glucose levels were measured before the injection. Insulin (1 U/kg) was then injected intraperitoneally according to body weight (0.75 μL/10 g, injection volume to body weight ratio). Tail blood glucose levels were measured at 15, 30, 60, and 120 min after injection (Wang et al., 2021).

### 2.6 Serum biochemical analysis

Blood was collected from the abdominal aorta of each rat and allowed to clot at room temperature for 4 h. The blood samples were then centrifuged at 3,500 rpm for 15 min at 4°C, after which the serum was carefully transferred to fresh centrifuge tubes for further analysis.

Serum levels of triglyceride (TG), cholesterol (CHO), and low-density lipoprotein (LDL) were measured using commercial assay kits following the manufacturer’s instructions. The assay conditions, including incubation times and temperatures, were strictly adhered to as per the kit protocols.

For hormone analysis, serum levels of testosterone (T), luteinizing hormone (LH), follicle stimulating hormone (FSH), insulin, and estradiol (E2) were quantified using specific enzyme-linked immunosorbent assay (ELISA) kits. Each ELISA assay was performed according to the manufacturer’s instructions. Absorbance was measured using a MicroplateReader at the specified wavelength for each hormone, and the concentrations were calculated based on standard curves generated for each assay.

### 2.7 Hematoxylin and eosin (H&E) staining

Fresh tissues were immediately fixed in 10% Paraformaldehyde (PFA) solution at room temperature for 24 h (Chen et al., 2022). After fixation, the tissues were dehydrated through a graded ethanol series (75% ethanol for 4 h, 85% ethanol for 2 h, 90% ethanol for 2 h, 95% ethanol for 1 h, and absolute ethanol twice for 30 min each). The tissues were then cleared in xylene twice for 5–10 min each.

Subsequently, the tissues were infiltrated with paraffin at 65°C, with three changes of paraffin, each lasting 1 h. The paraffin-infiltrated tissues were embedded and sectioned at a thickness of 4 μm. Sections were floated on a 40°C water bath, mounted on glass slides, and baked in a 60°C oven to ensure adherence.

For staining, sections were deparaffinized in xylene three times for 10 min each and rehydrated through 95% and 100% ethanol for 5 min each. Sections were stained with hematoxylin for 5 min, rinsed in tap water for 10 min, differentiated in 0.7% hydrochloric acid ethanol for 10 s, and blued in tap water for 10 min. Sections were then stained with eosin for 5 min, dehydrated in 95% ethanol and absolute ethanol (5 min each), cleared in xylene twice for 5 min each, and mounted with neutral resin.

### 2.8 16S rDNA sequencing

The fresh feces were collected from rats. DNA was extracted using the kits. The extracted genomic DNA was used as a template for PCR amplification of V3-V4 of 16S rDNA. Different samples of the same library were distinguished using upstream primers with Barcode tags. Libraries were constructed using the TruSeq Nano DNA LT Sample Preparation Kit and sequenced on an Illumina Miseq PE300 platform. The data2 plug-in of the QIIME2 platform was used to process data quality control. Species annotation of sequences was performed using the Silva database (version: SilvA-132-99). α-diversity was used to assess the richness and evenness of the microbiota, and β-diversity was used to evaluate the overall difference of the microbiota in different treatment groups. The ANOSIM index was used to compare the differences between and within groups. β diversity was characterized using PCoA, based on the Bray-Curtis distance between samples. Linear discriminant effect value analysis (LEfSe) was used to identify the characteristic flora of different treatment groups.

### 2.9 RNA-seq assay

Four livers from each group of rats were harvested for analysis. Total RNA was extracted and assessed. A cDNA library was constructed, and its quality was evaluated using an Agilent Bioanalyzer 2,100. The clustering of the index-coded samples was performed on a cBot Cluster Generation System using TruSeq PE Cluster Kit v3-cBot-HS (Illumina) according to the manufacturer’s instructions. After cluster generation, the library preparations were sequenced on an Illumina NovaSeq platform, and 150 bp paired-end reads. Differentially expressed genes were identified using SangerBOX (http://vip.sangerbox.com). Gene Ontology (GO) and Kyoto Encyclopedia of Genes and Genomes (KEGG) assays were used to evaluate the RNA-seq results.

### 2.10 Western blot assay

Total protein was extracted from ovarian tissue using RIPA lysis buffer containing 1× protease and 1× phosphatase inhibitors. Proteins were separated using SDS-PAGE electrophoresis system and transferred to PVDF membranes. 5% skim milk was used to block the membranes at 4°C overnight. After blocking, the membranes were incubated with the primary antibodies (diluted 1:1,000 in 5% skim milk/1× TBST) for 2 h at room temperature, washed with 1× TBST and then incubated with the secondary antibodies of the same source for 1 h. The membranes were subsequently washed with 1× TBST again and then visualized using an ECL detection kit. The protein Caspase-3 was identified, and the intensity of the protein bands was quantified using ImageJ software to determine grayscale values.

### 2.11 Immunohistochemical staining

The preparation of tissue sections was carried out according to the method described in Section 2.7. The sections were incubated in the dark with 3% hydrogen peroxide solution for 25 min to inhibit endogenous peroxidase activity. Following this, the sections were washed three times with PBS (pH 7.4), each wash lasting 5 min. Blocking was performed at room temperature using 3% BSA for 30 min. After blocking, the primary antibody was applied to the sections and incubated overnight at 4°C. The next day, the sections were washed three times with PBS, each wash lasting 5 min. The sections were then incubated with the secondary antibody at room temperature for 50 min, followed by three additional washes with PBS. The sections were stained with freshly prepared DAB solution. Counterstaining was performed with hematoxylin for 3 min, followed by rinsing with tap water, differentiation with hematoxylin differentiation solution, and a final rinse with tap water. Finally, the sections were treated with hematoxylin bluing solution, dehydrated through graded ethanol, cleared with xylene, and mounted. Quantitative analysis was performed using ImageJ software.

### 2.12 Data analysis

Statistical analysis was performed using SPSS (version 21) and GraphPad Prism 8 (GraphPad Software Inc., La Jolla, CA). Data were presented as the mean ± standard error of the mean (SEM). The normality of data distribution was checked using the Shapiro-Wilk test and K-S test. For data following a normal distribution, one-way analysis of variance (ANOVA) was used to compare differences between groups. For data not following a normal distribution, nonparametric tests were applied. A value of *P* < 0.05 was considered statistically significant.

## 3 Results

### 3.1 Letrozole and HFD induce PCOS-IR rats

**FIGURE 1:**
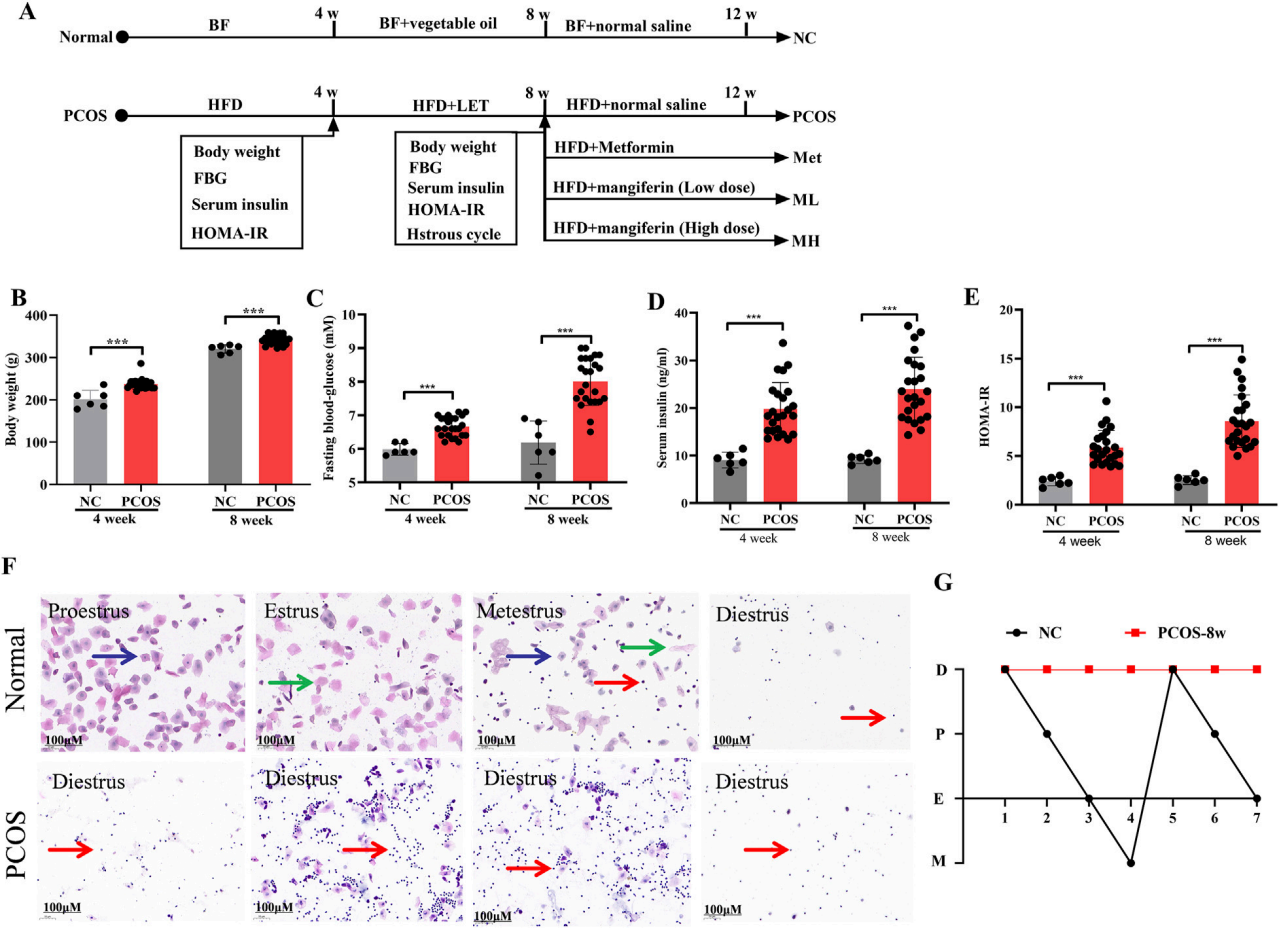
Letrozole and HFD induce PCOS-IR rats. **(A)** A schematic of the animal experiment. **(B)** The body weight of normal and PCOS rats. **(C)** The fasting blood-glucose of normal and PCOS rats. **(D)** The serum insulin of normal and PCOS rats. **(E)** The homeostatic model assessment-insulin resistance (HOMA-IR) of normal rats and PCOS rats. **(F)** Vaginal smears from different stages of the estrous cycle in the normal and PCOS rats. Blue arrows, nuclear epithelial cells; green arrows, cornified squamous epithelial cells; red arrows, leucocytes. **(G)** Linear chart of the of estrous cycle. Data are expressed as the mean ± SEM (NC group, n = 6; PCOS group, n = 24). ^*^ *p* < 0.05, ^**^ *p* < 0.01, ^***^ *p* < 0.001 vs. NC.

We used letrozole (LET) in combination with HFD to establish the rats model of PCOS with insulin resistance (PCOS-IR) (Figure 1A). Compared with normal rats (Figures 1B–E), model rats were kept with 4 weeks of HFD and injected with 4 weeks of LET (1 mg/kg/day), result in significant increases in fasting blood glucose (FBG), serum insulin, and homeostatic model assessment-insulin resistance (HOME-IR). Estrous cycle disorder is one of the key characteristics of polycystic ovary syndrome (PCOS) (Maliqueo et al., 2014). A typical estrous cycle comprises the proestrus, estrus, metestrus, and diestrus stages. As illustrated in Figure 1F, the proestrus phase is identified by the presence of nuclear epithelial cells (blue arrows). The estrus phase is mainly composed of cornified squamous epithelial cells (green arrows). The metestrus phase features leucocytes, nuclear epithelial cells, and cornified squamous epithelial cells, whereas the diestrus phase is dominated almost entirely by leucocytes (red arrows). Induction by LET + HFD disrupted the normal estrous cycle in rats, resulting in a prolonged diestrus phase and a significant increase in leucocytes. Based on these results, IR was significantly increased in model rats. In addition, the estrous cycle of model rats was in the diestrus stage, which was different from the estrous cycle of normal rats (Figure 1G). According to these results, the model rats of PCOS with IR were successfully reproduced.

### 3.2 Mangiferin could improve lipid metabolism disorder in PCOS-IR rats

**FIGURE 2:**
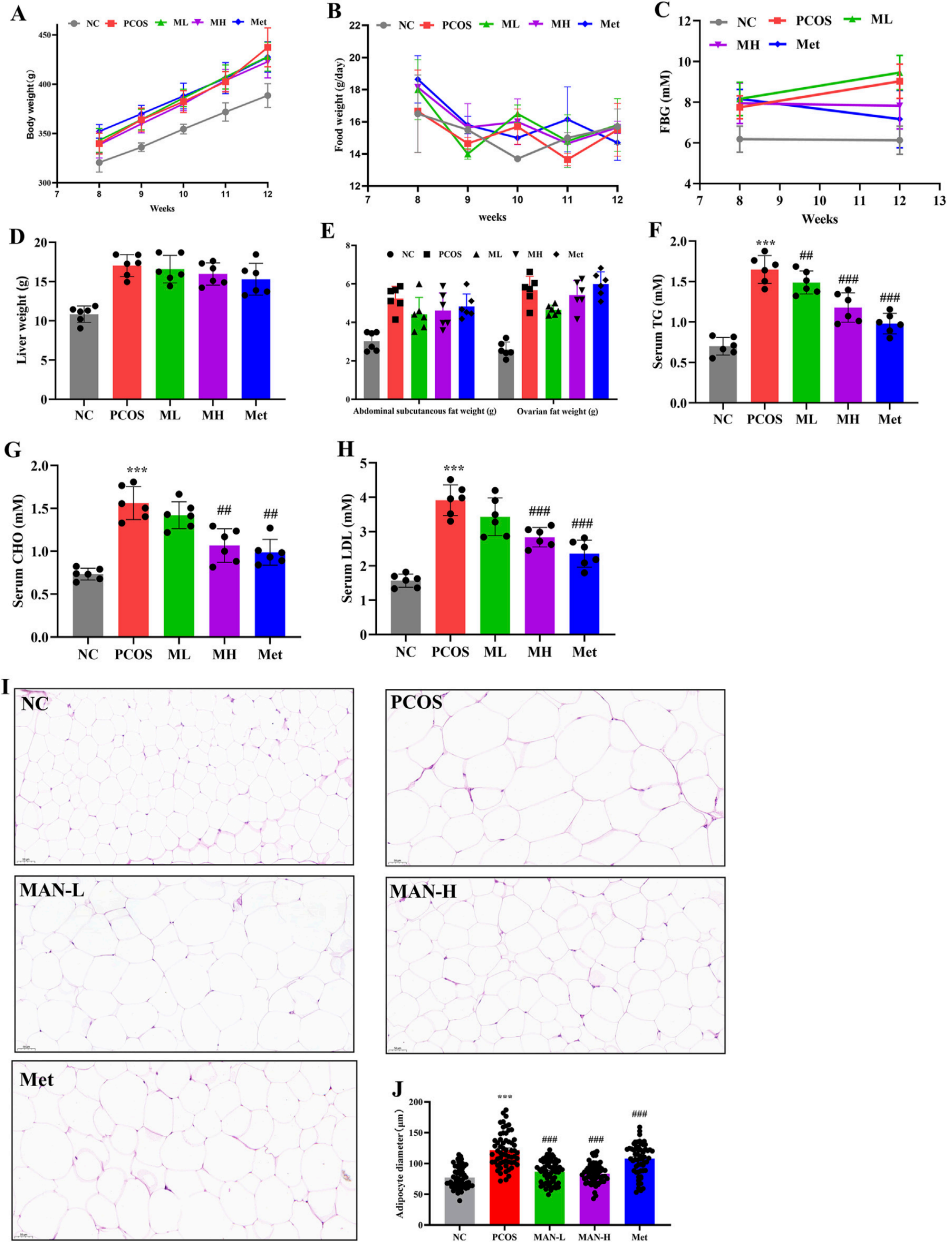
Mangiferin could improve lipid metabolism disorder in PCOS-IR rats. **(A)** Body weight. **(B)** Food intake. **(C)** Fasting blood glucose. **(D)** Liver weight. **(E)** Abdominal subcutaneous fat weight and ovarian fat weight. **(F)** Serum triglyceride (TG) levels. **(G)** Total cholesterol (CHO) levels. **(H)** Serum Low-density lipoprotein cholesterol (LDL) levels. **(I)** H&E staining of the adipocyte. **(J)** Adipocyte diameter. Data are expressed as mean ± SEM (n = 6 per group). *p < 0.05, **p < 0.01, ***p < 0.001 vs. NC; ^#^ *p* < 0.05, ^##^ *p* < 0.01, ^###^ *p* < 0.001 vs. PCOS.

To determine the effects of mangiferin on PCOS, we compared body weight, food intake, FBG, liver weight, abdominal subcutaneous fat weight, ovarian fat weight, serum TG, serum CHO, and serum LDL between different groups. Although mangiferin had no significant effects on body weight and food intake in PCOS rats. However, there was a significant decrease in FBG in the MH group compared to the PCOS group. This was similar to the Met group (Figures 2A–C). Mangiferin had no significant effects on liver weight, abdominal subcutaneous fat weight, and ovarian fat weight in PCOS rats (Figures 2D, E). However, mangiferin significantly and dose-dependently decreased the serum TG, serum CHO, and serum LDL in PCOS rats (Figures 2F–H). We also examined adipocyte abnormalities in the ovarian fat of rats across different groups, utilizing H&E staining. The diameter of adipocytes was measured to assess their size. Results indicated that adipocytes exhibited significantly larger sizes in PCOS rats compared to normal rats. Following mangiferin intervention, there was a significant reduction in adipocyte size (Figures 2I, J), aligning closely with the Met group. These results suggest that mangiferin could improve the lipid metabolism disorder of PCOS rats induced by the combination of HFD and LET.

### 3.3 Mangiferin ameliorates IR in PCOS rats

**FIGURE 3:**
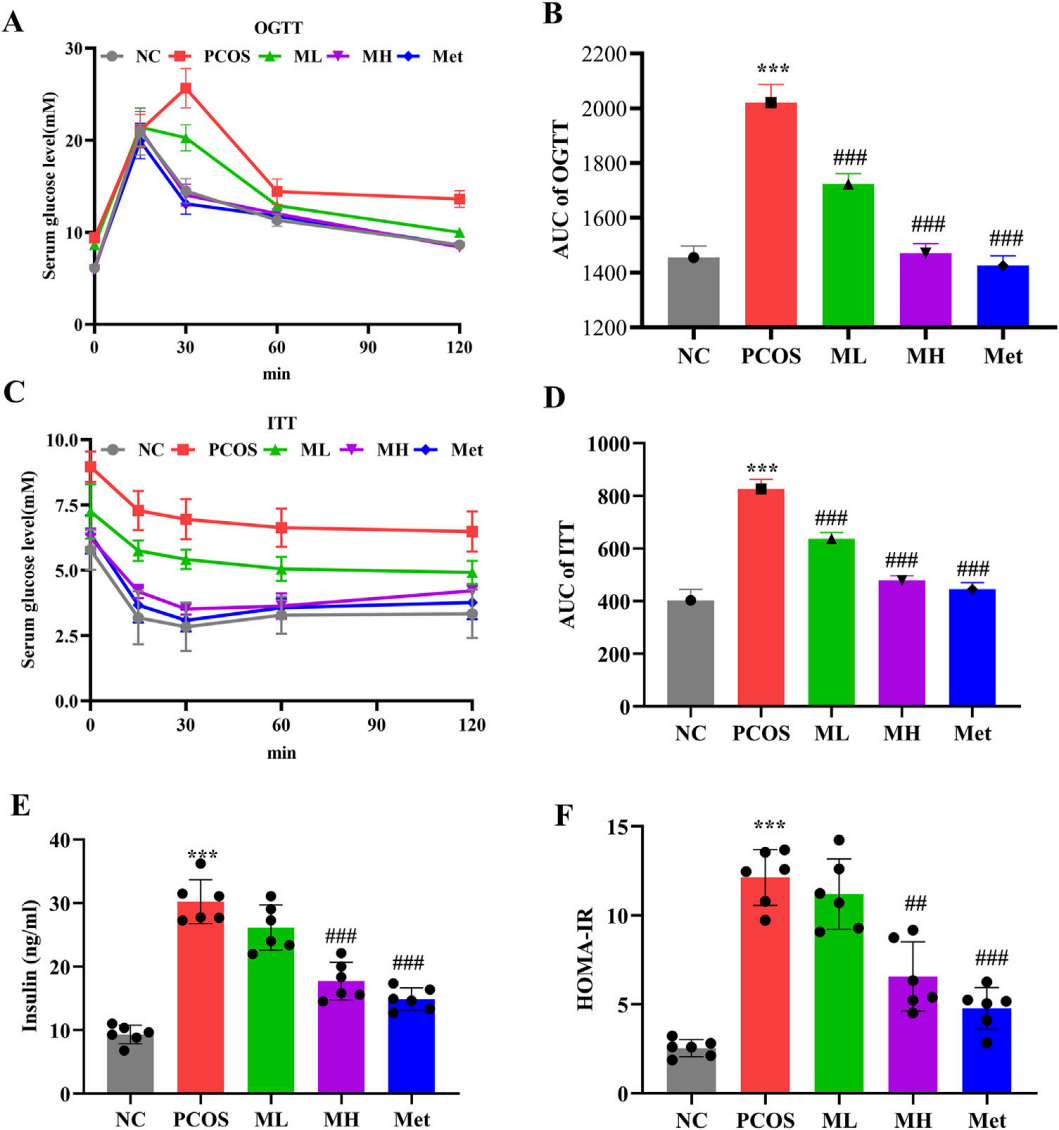
Mangiferin ameliorates IR in PCOS rats. **(A, B)** Oral glucose tolerance test (OGTT). **(C, D)** Insulin tolerance test (ITT). **(E)** Serum level of insulin. **(F)** HOMA-IR. Data are expressed as mean ± SEM. Data are expressed as mean ± SEM (n = 6 per group). *p < 0.05, **p < 0.01, ***p < 0.001 vs. NC; ^#^ *p* < 0.05, ^##^ *p* < 0.01, ^###^ *p* < 0.001 vs. PCOS.

IR induces hyperinsulinemia, which could exacerbate the symptoms of PCOS. We further investigated the effect of mangiferin on IR in PCOS rats. Compared with normal rats, glucose tolerance and insulin sensitivity were significantly elevated in PCOS rats, according to the area under the curve (AUC) of OGTT and ITT tests. However, mangiferin intervention could improve glucose tolerance and insulin sensitivity in PCOS rats (Figures 3A–D). In addition, the serum insulin level and HOMA-IR showed a significant increase in PCOS rats compared to normal rats, Mangiferin could reduce these indices (Figures 3E, F). These results suggest that mangiferin could ameliorate the IR in PCOS rats.

### 3.4 Mangiferin ameliorates hormonal imbalance in PCOS rats

**FIGURE 4:**
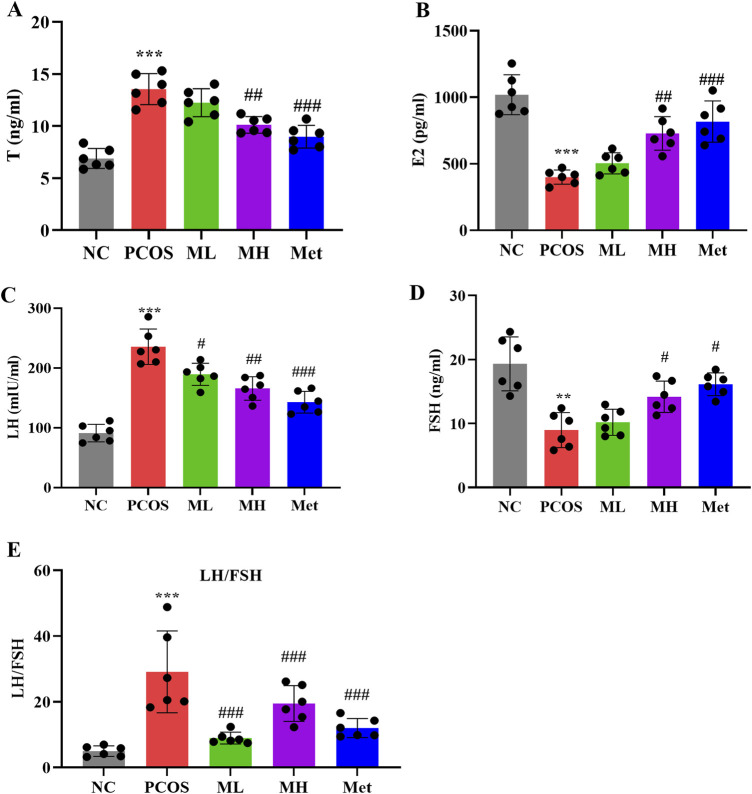
Mangiferin ameliorates hormonal imbalance in PCOS rats. **(A)** Serum levels of Testosterone (T). **(B)** Serum levels of estradiol (E2). **(C)** Serum levels of luteinizing hormone (LH). **(D)** Serum levels of follicle stimulating hormone (FSH). **(E)** LH/FSH ratio. Data are expressed as mean ± SEM (n = 6 per group). *p < 0.05, **p < 0.01, ***p < 0.001 vs. NC; ^#^ *p* < 0.05, ^##^ *p* < 0.01, ^###^ *p* < 0.001 vs. PCOS.

PCOS is characterized by hormonal imbalances. Compared with normal rats, serum T and LH levels were significantly increased, while serum FSH and E2 levels were significantly decreased in PCOS rats. The LH/FSH ratio, an important marker for PCOS detection, was notably elevated in PCOS rats (Figure 4E). After mangiferin intervention, the serum T and LH levels were decreased, and the serum FSH and E2 levels were increased. The LH/FSH ratio after magiferin intervention was markedly decreased (Figures 4A–E) Studies have shown that mangiferin can improve hormonal imbalances through the regulation of glucose and lipid metabolism and anti-inflammatory effects (Qian et al., 2020; Al-Saeedi, 2021). Hering et al. (2023) also indicated that mangiferin has potential effects in regulating reproductive hormones. These studies support our findings that mangiferin can improve hormonal imbalances in PCOS rats through multiple pathways, providing a novel and effective strategy for the treatment of PCOS.

### 3.5 Mangiferin can improve ovarian function in PCOS rats

**FIGURE 5:**
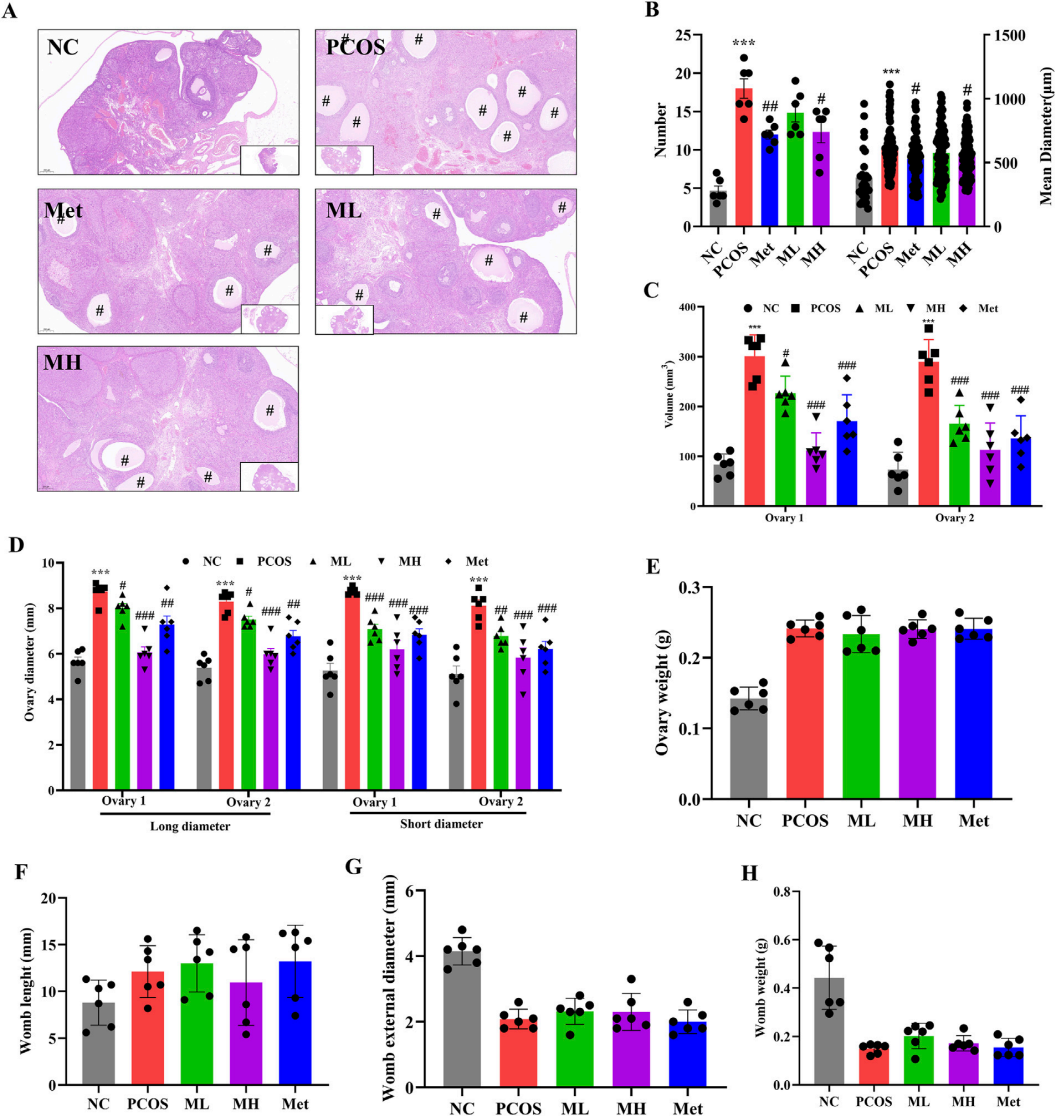
Mangiferin can improve ovarian function in PCOS rats. **(A)** H&E staining of the ovaries; **(B)** The number and mean diameter of ovarian cysts in each sample. **(C)** The volume of the ovaries. **(D)** The long and short diameter of ovaries; **(E)** The weight of ovaries; **(F)** The length of womb; **(G)** The external diameter of womb; **(H)** The weight of womb. Data are expressed as mean ± SEM (n = 6 per group). “#” indicated the cystic follicles. *p < 0.05, **p < 0.01, ***p < 0.001 vs. NC; ^#^ *p* < 0.05, ^##^ *p* < 0.01, ^###^ *p* < 0.001 vs. PCOS.

Ovarian and womb morphological defects often accompany PCOS. We assessed the effects of mangiferin on ovarian and womb morphology in PCOS rats. Compared with normal rats, the size of follicles and their number were increased in PCOS rats (Figures 5A, B), and the diameter and volume of ovaries were increased in PCOS rats (Figures 5C, D). The number and size of cavities in the ovarian of PCOS rats were significantly reduced after mangiferin intervention (Figures 5A, B). However, mangiferin had no significant effects on the ovary weight, the womb length, womb external diameter and womb weight in PCOS rats (Figures 5E–H). The above results suggest that mangiferin could improve ovarian function in PCOS rats.

### 3.6 Mangiferin alters the diversity of gut microbiota in PCOS rats

**FIGURE 6:**
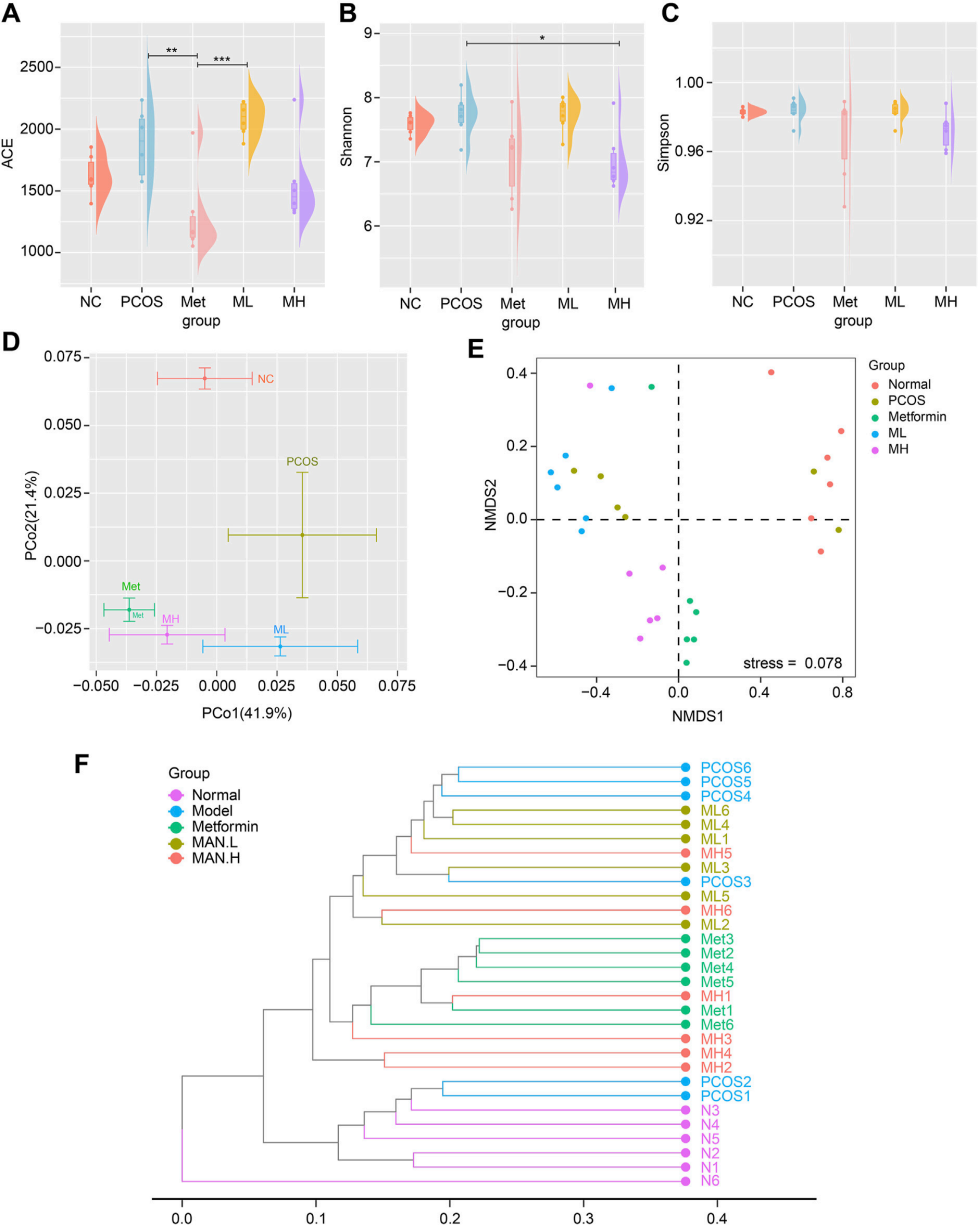
Mangiferin alters the diversity of gut microbiota in PCOS rats. α-diversity measured by **(A)** ACE index, **(B)** Shannon index, and **(C)** Simpson index. **(D)** β-diversity measured by PCoA. The gut microbiota composition of **(E)** NMDS2 analysis, **(F)** Dendrogram analysis.

Several studies have shown that PCOS alters the composition of gut microbiota. We used 16S rDNA gene sequencing to assess whether mangiferin could improve gut microbiota. Shannon index indicated a significant difference in α diversity between the PCOS and MH groups (Figures 6A–C). PCoA analysis revealed that the PCOS group was completely different from the NC group. The ML group, MH group and Met group were completely different from the PCOS group and close to each other in β diversity (Figure 6D). These results indicated that mangiferin could alter the gut microbiota of PCOS, which was similar to the effect of Met group in β diversity. NMDS analysis showed a significant difference in gut microbiota diversity between the NC group and PCOS group. At the same time, there was no a significant difference between the ML group and the PCOS group. The gut microbiota composition of the MH group was similar to that of the Met group and different from that of the PCOS group (Figure 6E). The dendrogram analysis showed similar results to NMDS: the MH group was different from the PCOS group, while close to the Met group (Figure 6F). These results suggest that high-dose mangiferin (MH) may alter gut microbiota diversity in PCOS rats, which is similar to the Met group.

### 3.7 Mangiferin alteres the composition of gut microbiota in PCOS rats

**FIGURE 7:**
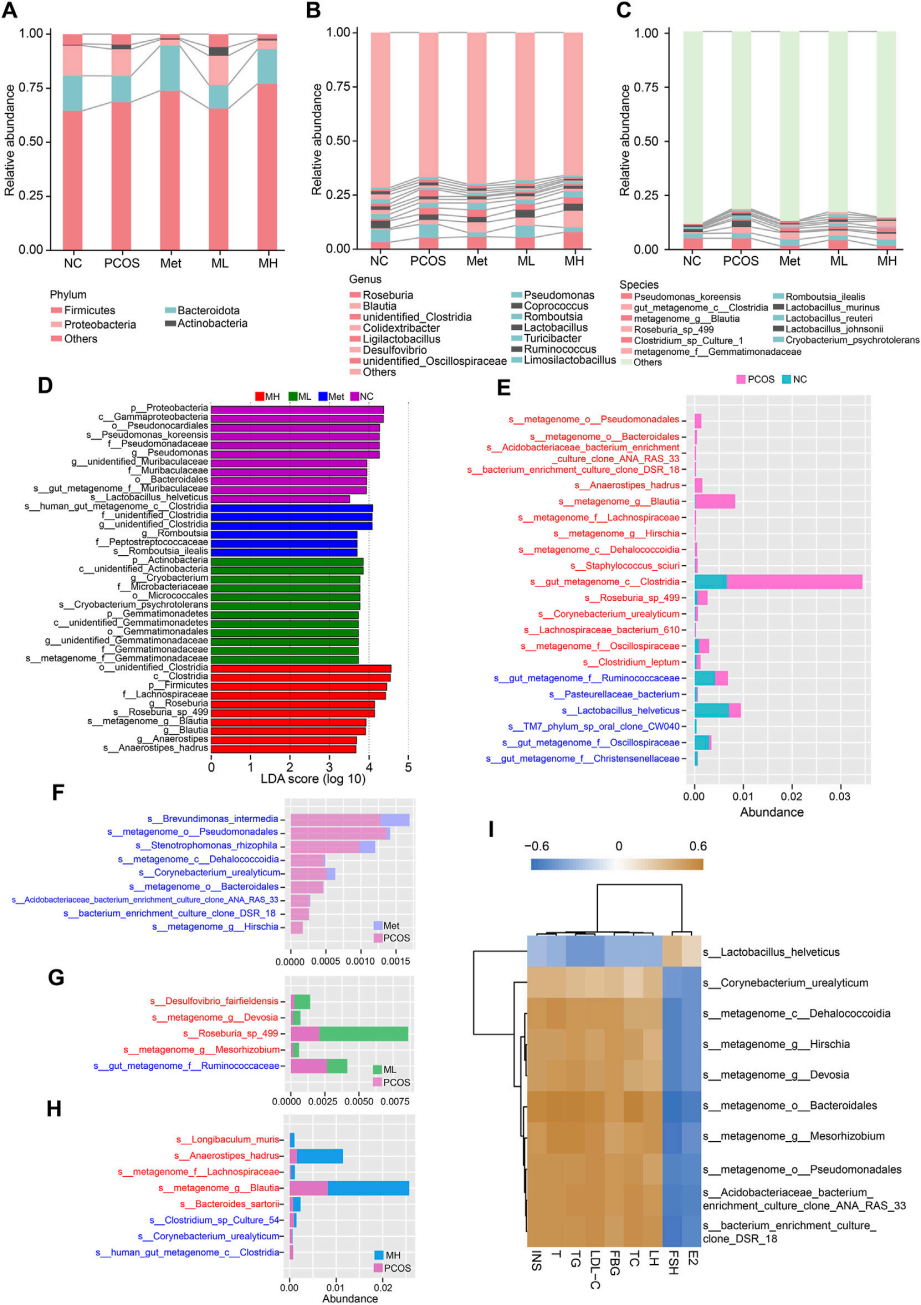
Mangiferin alteres the composition of gut microbiota in PCOS rats. **(A)** Relative abundance of gut microbiota at the phylum level, **(B)** at the genus level, and **(C)** at the species level. **(D)** Histogram of LEfSe analysis. Histogram comparisons for gut microbiota composition in different groups: **(E)** The PCOS and NC groups, **(F)** The Met and PCOS groups, **(G)** The ML and PCOS groups, **(H)** The MH and PCOS groups, **(I)** Heatmap of the relationship between gut microbiota and hormone levels.

Quantitative analysis of the gut microbiota revealed an increased relative abundance of actinobacteria and firmicutes. In contrast, the relative abundance of bacteroidota and proteobacteria decreased in the PCOS group compared with the NC group at the phylum level. A low dose of mangiferin (ML group) did not alter this pattern. In contrast, a high dose of mangiferin (MH group) increased the relative abundances of firmicutes and bacteroidota and decreased those of proteobacteria and actinobacteria, consistent with metformin (Figure 7A). At the genus level, the relative abundance of *Roseburia*, *Blautia*, *unidentified Clostridia,* and *Ligilactobacillus* were increased in the PCOS group compared with the NC group. In contrast, the relative abundance of *Coprococcus*, *Lactobacillus*, *Turicibacter* and *Desulfovibrio* were decreased. The relative abundance of *Blautia* and *Coprococcus* were increased in the MH group compared with the PCOS group. Similar to the Met group, high-dose mangiferin increased the relative abundance of *Roseburia* and *Blautia*, and decreased the relative abundance of *Pseudomonas* compared with the PCOS group (Figure 7B). At the species level, the relative abundance of *gut_metagenome_c__Clostridia*, *Lactobacillus_murinus*, *metagenome_g_Blautia*, *Roseburia_sp_*499, *Clostridium_sp_Culture*-1, *Cryobacterium_psychrotolerans* and *metagenome_f_Gemmatimonadaceae* were increased in the PCOS group compared to the NC group. A low-dose mangiferin increased the relative abundance of *Cryobacterium_psychrotolerans* and decreased the relative abundance of *Lactobacillus_murinus* compared to the PCOS group. A high-dose mangiferin increased the relative abundance of *Romboutsia_ilealis* and *Roseburia_sp_*499, and decreased the relative abundance of *Pseudomonas_koreensis*, *Lactobacillus_murinus*, *Lactobacillus_reuteri* and *metagenome_f__Gemmatimonadaceae* compared to PCOS group, similar to the Met group (Figure 7C). To identify the dominant bacterias in each group, we performed linear discriminant analysis and Effect Size (LEfSe) analysis. No significantly dominant bacterias were found in the PCOS group. There were many dominant bacterias in the NC group, with *p__Proteobacteria* and *c__Gammaproteobacteria* possessing the highest relative abundance. The mainly dominant bacterias in the Met group were several species of *Clostridia* The mainly dominant bacterias in the ML group were several species of *Gemmatinonadetes*. The abundance of *p__Firmicutes*, *c__Clostridia* and *f__Lachnospiraceae* was relatively high in the MH group (Figure 7D). In a pairwise comparison, we found that there was a significantly increased abundance of *s__metagenome_g__Blautia* and *s__gut_metagenome_c__Clostridia* in the PCOS group, while there was modestly increased abundance of *s__metagenome_o__Pseudomonadales*, *s__Anaerostipes_hadrus*, *s__Roseburia_sp_498*, and *s__metagenome_f__Oscillospiraceae* and others (Figure 7E). The abundance of *s__Brevundimonas_intermedia*, *s__metagenome_o__Pseudomonadales*, *s__Stenotrophomonas_rhizophila* and others was increased in the Met group compared with the PCOS group (Figure 7F). The abundance of *s__Desulfovibrio_fairfieldensis*, *s__metagenome_g__Devosia*, *s__Roseburia_sp_*499, *s__metagenome_g__Mesorhizobium*, and *s__gut_metagenome_f__Ruminoccoccaceae* was increased in the ML group compared with the PCOS group (Figure 7G). The comparison between the PCOS and MH groups showed that the abundance of *s__Anaerostipes_hadrus* and *s__metagenome_g__Blautia* was markedly increased. In addition, the abundance of *s__Longibaculum_muris*, *s__metagenome_f__Lachnospiraceae*, *s__Bacteroides_sartoril* and *s__Clostridium_sp_Culture_54* was increased in the MH group (Figure 7H). The correlation analysis revealed a positive association between the relative abundance of *s__Lactobacillus_helveticus* and the FSH and E2 levels, while a negative association between the relative abundance of *s__Lactobacillus_helveticus* and the serum levels of INS, T, TG, LDL-C, FBG, TC, and LH. In contrast, the relative abundance of *s__Corynebacterium_urealyticum*, *s__metagenome_c__Dehalococcoidia*, *s__metagenome_g__Hirschia*, *s__metagenome_g__Devosia*, *s__metagenome_o__Bacteroidales*, *s__metagenome_g__Mesorhizobium*. *S__metagenome_o__Pseudomonadales*, *s__Acidobacteriaceae_bacterium_enrichment_culture_clone_ANA_RAS*_33 and *s__bacterium_enrichment_culture_clone_DSR_*18 was positively correlated with the serum level of INS, T, TG, LDL-C, FBG, TC, and LH, while negatively correlated with the serum level of FSH and E2 (Figure 7I). These results suggested that changes in the abundance of *lactobacillus* may be associated with mangiferin-mediated improvement in hormonal imbalance.

### 3.8 Correlation analysis of gut microbiota and functional prediction

**FIGURE 8:**
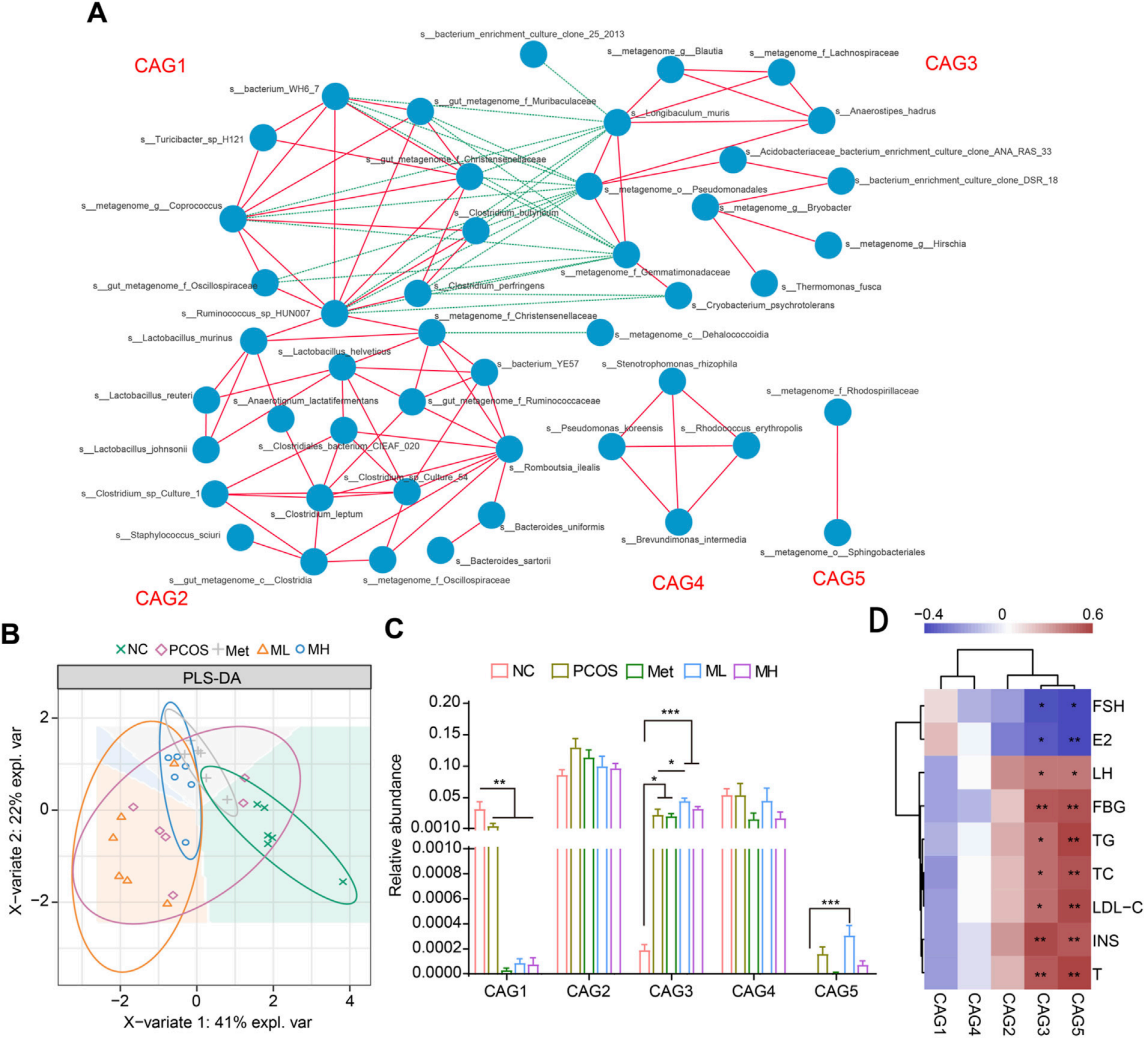
Correlation analysis of gut microbiota and functional prediction. **(A)** Gut microbiota was divided into five groups. **(B)** PLA-DA analysis. **(C)** Relative abundance of different groups. **(D)** Heatmap of the relationship between gut microbiota and hormone. Data are expressed as mean ± SEM (n = 6). ^*^ *p* < 0.05, ^**^ *p* < 0.01, ^***^ *p* < 0.001 vs. NC or PCOS.

We performed a correlation network analysis to determine whether the therapeutic effects of mangiferin were related to the gut microbiota. Based on the relationships between gut microbiota, we divided them into five groups, from CAG1 to CAG5 (Figure 8A). CAG1 was complex and composed of *Bilophila*, *Coprococcus*, *Oscillospira*, *Ruminococcus* and others. CAG2 was mainly composed of *Lactobacillus*. CAG3 was composed of several species of *Lachnospiraceae* and *Gemmatimonadaceae*. CAG4 was composed of *s__Pseudomonas_koreensis*, *s__Rhodococcus_erythropoils*, *s__Stenotrophomonas_rhizophila* and *s__Brevundimonas_intermedia*. CAG5 was composed of the *s__ metagenome_f_Rhodospirillaceae* and *s__metagenome_o__Sphingobacteriales*. The PLA-DS analysis showed that mangiferin did not significantly alter the distribution of gut microbiota species (Figure 8B). However, in the group level, mangiferin significantly increased the abundance of gut microbiota in the CAG3 and CAG5 groups and significantly decreased the abundance of gut microbiota in the CAG1 group (Figure 8C). LH, FBG, TG, TC, LDL-C, INS, and T were correlated with the CAG3 and CAG5 groups, while FSH and E2 were strongly and negatively correlated with these groups (Figure 8D). These results partially explain why mangiferin could improve hormonal imbalance and IR.

### 3.9 KEGG pathway prediction for the effects of mangiferin on gut microbiota

**FIGURE 9:**
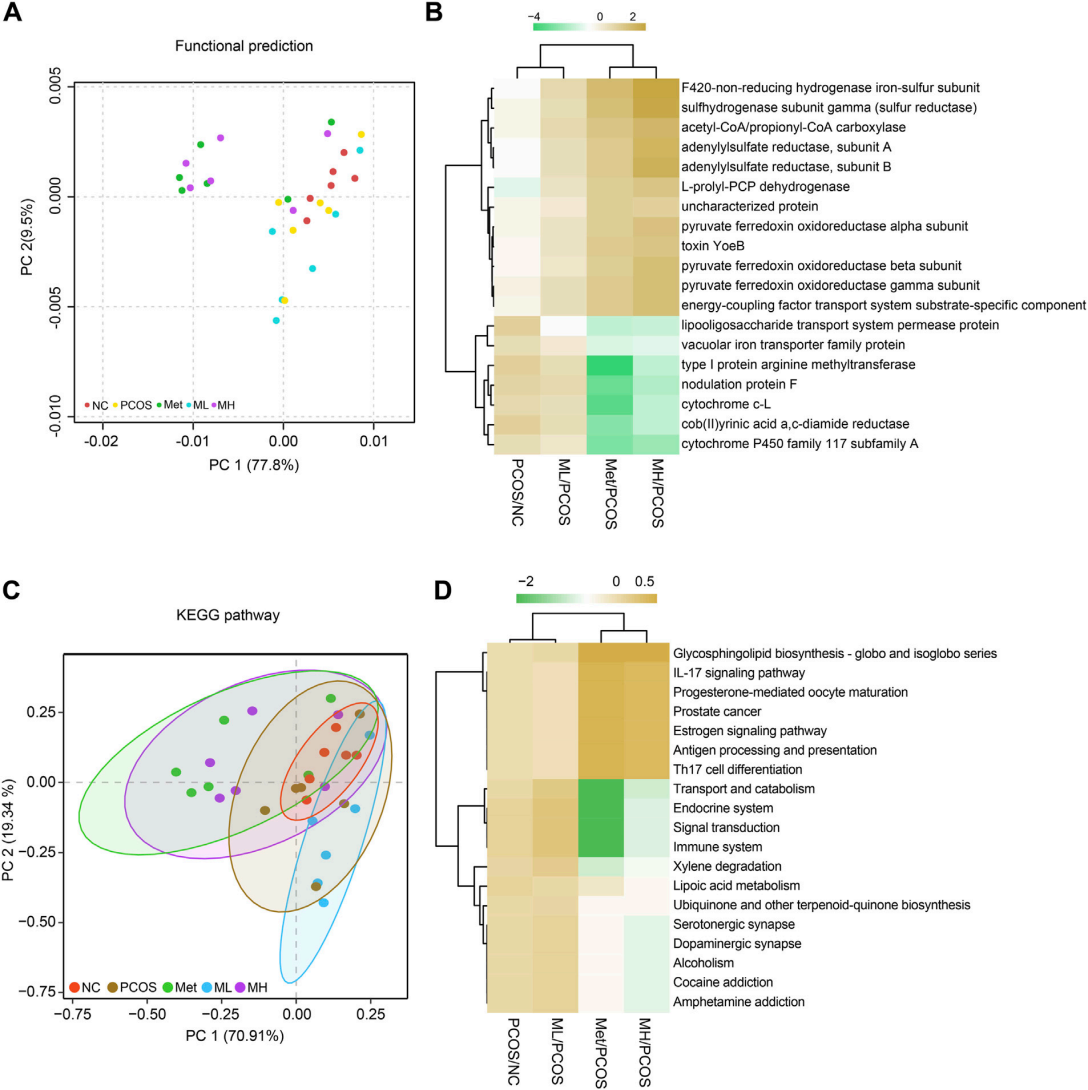
KEGG pathway predicting the effects of mangiferin on gut microbiota. **(A, B)** functional predictions, **(C, D)** KEGG pathway predictions.

We predicted the effects of mangiferin on gut microbiota in PCOS rats. The results showed that mangiferin was involved in several metabolic and inflammatory pathways. Mangiferin was positively correlated with PCOS in many metabolic function enzymes, such as the F420 non-reducing hydrogenase iron-sulphur subunit, sulfhydrogenase subunit gamma and adenylylsulfate reductase (Figures 9A, B). In addition, mangiferin was involved in glycosphingolipid biosynthesis, IL-17 signalling pathway and others (Figures 9C, D). The results indicate that mangiferin may play a significant role in these physiological functions associating with metabolic and inflammatory by regulating gut microbiota.

### 3.10 RNA-Seq assay of the effects of mangiferin on PCOS

**FIGURE 10:**
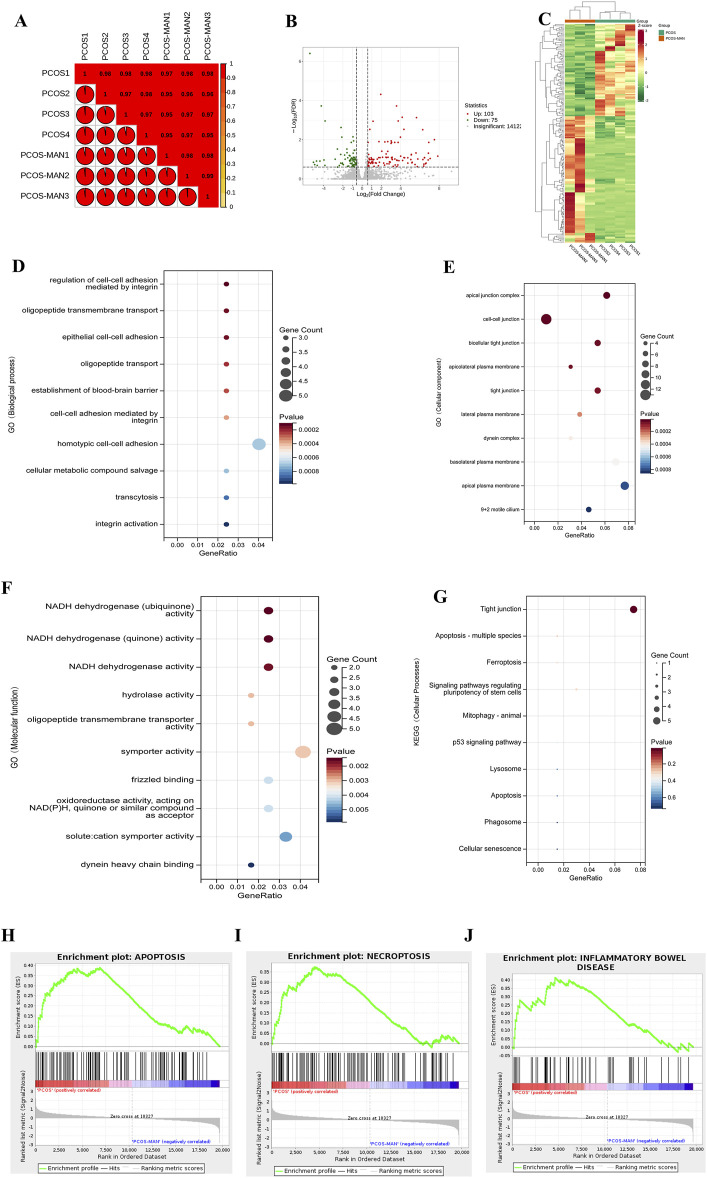
RNA-Seq assay of the effects of mangiferin on PCOS. **(A)** Differential rates of gene expression in PCOS before and after intervention with mangiferin. **(B)** Volcanic map analysis of differential gene expression before and after treatment with mangiferin. **(C)** Heatmap of the overall differential gene expression. GO analysis of gene function in **(D)** biological process, **(E)** cellular component, **(F)** molecular function and **(G)** cellular processes. GSEA analysis of PCOS and **(H)** mangiferin in apoptosis, **(I)** necroptosis and **(J)** inflammatory bowel disease.

To further clarify the effects of mangiferin on PCOS, we conducted the RNA-seq of ovarian tissue. The Pearson R2 values of all samples were over 0.95, suggesting good repeatability (Figure 10A). In total, 178 differentially expressed genes (|log_2_(fold change)| > 1 and corrected *p-*value (padj) ≤0:05) were found using the DESeq2 R package (1.20.0) and visualized with volcano plot (Figure 10B), in which 103 genes were upregulated, and 75 genes were downregulated (Figure 10B). The heat map was also used to visualize differentially expressed genes (Figure 10C). GO analysis showed that mangiferin significantly modulated cell-cell adhesion and junction between ovarian cells (Figures 10D, E). In terms of molecular function, GO analysis showed that the therapeutic effects of mangiferin may be significantly associated with NADH dehydrogenase activity (Figure 10F). In the KEGG analysis, mangiferin affected ovarian cell apoptosis and tight junctions (Figure 10G). Gene Set Enrichment Analysis (GSEA) analysis indicated that apoptosis, necroptosis, and inflammatory bowel disease-associated gene sets were significantly upregulated in the ovarian tissue of PCOS rats, and mangiferin decreased the enrichment score (ES) of these gene sets (Figures 10H–J).

### 3.11 Mangiferin ameliorates the ovarian cell apoptosis in PCOS rats

**FIGURE 11:**
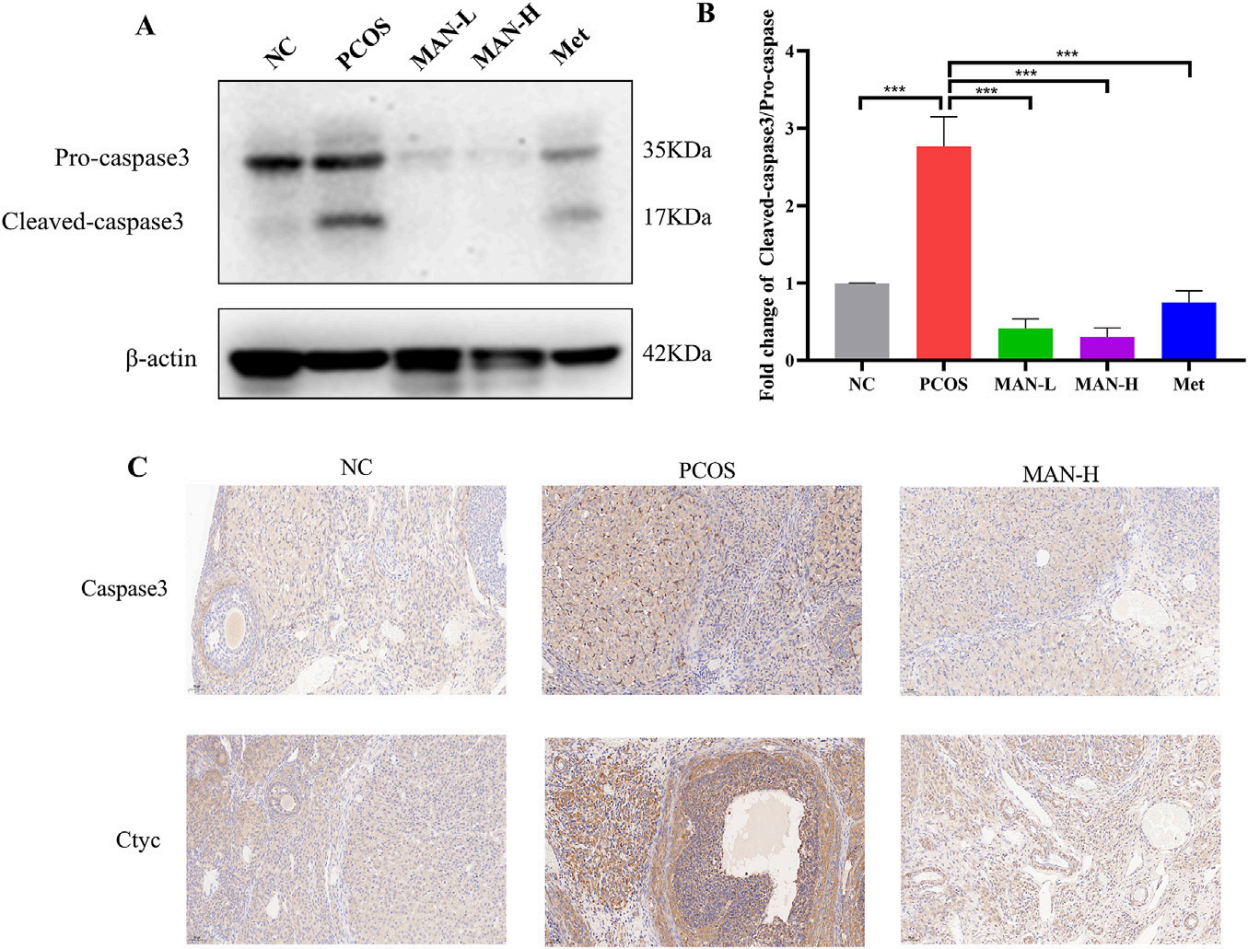
Mangiferin ameliorates the ovarian cell apoptosis in PCOS rats. **(A)** Examples of representative blots. **(B)** Fold changes of Cleaved-caspse3/Pro-caspase3, Data are expressed as mean ± SEM (n = 6). *p < 0.05, **p < 0.01, ***p < 0.001 vs NC or PCOS. **(C)** Representative immunohistochemical staining images of ovarian tissue for each group.

We further investigated the protein levels of Caspase3, which are associated with cell apoptosis. Western blot revealed that mangiferin significantly decreased the level of cleaved-caspase3, as well as the cleaved-caspase3/pro-caspase3 ratio, compared with the PCOS group (Figures 11A, B). Additionally, immunohistochemical staining demonstrated that mangiferin significantly reduced the PCOS-induced abnormal elevations of Caspase3 and Cyt c (Figure 11C). These results suggest that mangiferin can ameliorate the ovarian cell apoptosis.

## 4 Discussion

As a complex endocrine and metabolic disorder, PCOS is characterized by heterogeneous etiologies and clinical phenotypes (He et al., 2021). Various factors contribute to the development of PCOS, including insulin resistance, high serum androgen levels, air pollution, and genetic mutations (Zhang et al., 2020), which often interact with each other (Kumar et al., 2022). The complex and unclear pathogenesis and diverse clinical phenotypes make the cure of PCOS challenging. And there is an urgent need to develop new drug for PCOS treatment.

Hyperandrogenism, a classic symptom of hormonal imbalance in PCOS, is considered a primary cause and key factor in its development. In normal conditions, androgen release from theca cells by LH triggering, which production is stimulated by GnRH. Androgens further promote the growth of preantral and small antral follicles, and upregulate AMH, which activates GnRH-releasing neurons. FSH is a critical factor for hormone homeostasis, which can convert androgens into estradiol. Increasing levels of estradiol triggers ovulation, decreases GnRH production (Emanuel et al., 2022) and stimulates pre-antral follicle growth. In PCOS, excessive androgens can increase AMH and GnRH production. GnRH further promotes LH production over FSH and leads an increasing LH/FSH ratio. It is worse that increasing LH promotes theca cells to produce androgens and exacerbates hormonal disorders (Bulsara et al., 2021; Ashraf et al., 2019). Decreasing androgen level in serum and improving hormonal imbalance are essential to PCOS treatment. Oral contraceptives, such as drospirenone, norgestimate, cyproterone acetate, are commonly used to decrease androgen level in serum by affecting the steroidogenesis of hypothalamus, pituitary and ovarian (Rashid et al., 2022; Manzoor et al., 2019; Xing et al., 2022). But oral contraceptives can seriously affect the fertility of PCOS patients (Luque-Ramírez, et al., 2020). In this study, we found that mangiferin, a nature compound from *Mangifera indica* L., could reduce the androgens level of serum and improve hormonal imbalance in PCOS rats.

Insulin resistance (IR) stands out as a primary contributor to PCOS, with enhancing insulin sensitivity emerging as a crucial strategy in its treatment (Genazzani, 2020). IR prompts an elevation in serum insulin level, leading to hyperinsulinemia. Notably, insulin sensitivity remains unaffected in certain tissues like the ovaries, pituitary gland, and adrenal glands, which instead become overstimulated by the compensatory elevation in insulin. Within the ovaries, insulin collaborates with LH to stimulate androgen production in the ovarian cells (Moghetti and Tosi, 2021), and exacerbate hyperandrogenism (Janssen, 2022). In our study, mangiferin significantly reduced serum insulin level and HOMA-IR in PCOS rats, suggesting its potential benefit in PCOS.

The gut microbiota plays a crucial role in the development of PCOS as highlighted by many researchers. Recently studies elucidated significant differences in gut microbiota composition between healthy individuals and those with PCOS (Gu et al., 2022). Both 16S rRNA and metagenomic gene sequencing analysis have unveiled changes in α and β diversity among PCOS patients, along with notable alterations in the abundance of Bacteroidobacteria and Firmicutes (Zhou et al., 2020). Various factors contribute to these differences, including levels of hormone, insulin resistance, and obesity (Kshetrimayum et al., 2019). Changes in gut microbiota composition within PCOS individuals may disrupt intestinal barrier integrity, metabolic processes, and inflammatory responses (Moser et al., 2022; Sugawara et al., 2022). Metabolites produced by the gut microbiota, such as secondary bile acids, short-chain fatty acids (SCFAs), and trimethylamine (TMA), can exert direct effects on the intestines or enter systemic circulation, impacting various tissues including the ovaries, liver, skeletal muscle, and adipose tissue. These metabolites are associated with hyperandrogenism, insulin resistance, chronic inflammation, and abnormal levels of brain-gut peptides in PCOS (Li et al., 2022). In certain studies, transplantation of gut microbiota from healthy rats into PCOS rats resulted in improvements in their reproductive cycles, ovarian pathology, and regulation of hormone (Hamamah et al., 2022). Remodeling the composition of gut microbiota and modulating their metabolites have been identified as effective strategies for improving the development of PCOS. In our study, mangiferin can remodel the composition of gut microbiota in PCOS rats, and is associated with FSH, E2, LH and T, which partly explains the effects of mangiferin on hormonal homeostasis.

## 5 Conclusion

Our study demonstrated that mangiferin alleviates PCOS symptoms induced by LET combined with HFD in rats. This effect is primarily due to its ability to improve hormonal imbalances, insulin resistance, gut microbiota dysbiosis, and ovarian cell apoptosis. These findings suggest that mangiferin holds promise as a potential treatment for PCOS.

## Data Availability

The 16S rDNA data presented in the study are deposited in the NCBI repository, accession number: PRJNA1160415. Available at: http://www.ncbi.nlm.nih.gov/bioproject/1160415.
